# Society Meetings

**Published:** 1898-07

**Authors:** 


### SOCIETY MEETINGS.

The Medical Society of the County of Erie held a special meeting, Thursday evening, June 23, 1898, to consider the propriety of taking some action concerning the status of the nursing-bottle ordinance. The vice-president, Dr. J. B. Coakley, called the meeting to order and stated its object. Dr. H. R. Hopkins offered the following declaration of principles and resolutions :

Whereas, The Medical Society of the County of Erie, in common with the profession of medicine and other well-informed and high-minded persons, maintain the following principles, to-wit :

That the preservation of the public health is the first duty of the state ;

That the prevention of the communicable diseases is the plain and imperative duty of the sanitarian ;

That the responsibility of the state to afford protection to its citizens increases with the ignorance, carelessness and helplessness of such citizens ;

That of all our objects of protection, none are more helpless or in more constant need of wise and discriminating care than the newly-born, who must be reared by artificial feeding ;

That the death-rate among the artificially-fed newly-born marks the level of the sanatory enlightenment of the community ; and

Whereas, Competent medical opinion has and does unhesitatingly and unanimously condemn a certain type of feeding bottles in frequent use ; and

Whereas, Inspired by such well-founded conviction, ordinances forbidding the sale of this apparatus were recently enacted by the proper authorities of the city ; therefore

*Resolved*, That we approve heartily and in the most unqualified terms of the ordinance prohibiting the sale of any bottle that has connected therewith a rubber tube, hose or similar contrivance ;

*Resolved*, That as the relations between the professions of medicine and pharmacy should be most reciprocal and harmonious we commend to the druggists and pharmacists of our city our health ordinances in general and with greater particularity the ordinance forbidding the sale of a certain class of feeding bottles as timely, wise and in the best interests of the public health and that we invite them to join us in supporting these ordinances with their most earnest and constant efforts ;

*Resolved*, That we commend to all those in authority the subject of the preservation of the lives of the newly-born and those of tender years as of the highest practical importance and deserving of their incessant and sleepless vigilance.

Dr. Hopkins supported the foregoing in a timely speech that abounded in well-chosen sentences. After further remarks by Drs. Snow, Bayliss, W. D. Greene, Potter, Ring, the president, Dr. Lucien Howe and others, the preamble and resolutions were adopted.

---

THE Medical Society of the County of Chautauqua will hold its annual meeting at the Hotel Atheneum, Chautauqua, N. Y., Tuesday, July 12, 1898, at 11 o'clock a. m. The program is as follows: (1) Election of officers; (2) President's annual address; (3) Case of secondary hemorrhage, Dr. M. N. Bemus, Jamestown; General discussion; (4) Report or presentation of interesting cases; (5) Paper, Duodenitis, Dr. V. M. Griswold, Fredonia ; (6) Paper, Appendicitis, Dr. J. J. Mahoney, Jamestown ; Discussion, Dr. T. E. Soules, Cherry Creek and Dr. Geo. F. Smith, Sinclairville ; (7) Paper, Brain Tumor, Dr. Wm. C. Krauss, Buffalo ; Discussion, Dr. L. Hazeltine, Jamestown and Dr. L. C. Green, Panama ; (8) Paper, Early diagnosis of malignancy in abdominal and pelvic disease, Dr. C. C. Frederick, Buffalo ; (9) Paper, Dr. Wm. Seman Bainbridge, New York City. The annual election of officers will take place before dinner. There will be an evening session of scientific work to which all are earnestly requested to remain.

---

THE Thomas J. King Medical Club is the name given to a new organisation that was perfected at Delavan, Cattaraugus Co., N. Y., June 18, 1898. Its membership will include physicians residing in the northeastern towns of Cattaraugus and adjoining towns in Erie, Wyoming and Alleghany counties. Meetings will be held in June,

August, October, January and April, at places to be designated at the previous meeting. Its next meeting will be held at Springville, August 11, 1898. The officers are: President, Dr. Clarence King, of Machias; vice-president, Dr. William H. Jackson, of Springville; Secretary and treasurer, Dr. W. J. C. Evans, of Farmersville Station. At the initial meeting Dr. King read a paper on Dyspepsia.

